# Diogenes syndrome in dementia: a case report

**DOI:** 10.1192/bjo.2020.171

**Published:** 2021-02-02

**Authors:** Luca Sacchi, Emanuela Rotondo, Sara Pozzoli, Alessio Fiorentini, Giuseppina Schinco, Clara Mandelli, Carlotta Coppola, Giorgio G. Fumagalli, Tiziana Carandini, Anna M. Pietroboni, Daniela Galimberti, Fabio Triulzi, Giorgio Marotta, Elio Scarpini, Matteo Cesari, Paolo Brambilla, Andrea Arighi

**Affiliations:** Dino Ferrari Center, University of Milan, Italy; Neurodegenerative Diseases Unit, Fondazione IRCCS Ca’ Granda Ospedale Maggiore Policlinico, Milan, Italy; Department of Neurosciences and Mental Health, Fondazione IRCCS Cà Granda Ospedale Maggiore Policlinico, Milan, Italy; Department of Neurosciences and Mental Health, Fondazione IRCCS Cà Granda Ospedale Maggiore Policlinico, Milan, Italy; Geriatric Unit, Fondazione IRCCS Ca' Granda Ospedale Maggiore Policlinico, Milan, Italy; Geriatric Unit, Fondazione IRCCS Ca' Granda Ospedale Maggiore Policlinico, Milan, Italy; Geriatric Unit, Fondazione IRCCS Ca' Granda Ospedale Maggiore Policlinico, Milan, Italy; Neurodegenerative Diseases Unit, Fondazione IRCCS Ca' Granda Ospedale Maggiore Policlinico, Milan, Italy; Neurodegenerative Diseases Unit, Fondazione IRCCS Ca' Granda Ospedale Maggiore Policlinico, Milan, Italy; Neurodegenerative Diseases Unit, Fondazione IRCCS Ca' Granda Ospedale Maggiore Policlinico, Milan, Italy; Neurodegenerative Diseases Unit, Fondazione IRCCS Ca' Granda Ospedale Maggiore Policlinico, Milan, Italy; and Dino Ferrari Center, University of Milan, Italy; Neuroradiology Unit, Fondazione IRCCS Ca' Granda Ospedale Maggiore Policlinico, Milan, Italy; Nuclear Medicine Unit, Fondazione IRCCS Ca' Granda Ospedale Maggiore Policlinico, Milan, Italy; Neurodegenerative Diseases Unit, Fondazione IRCCS Ca' Granda Ospedale Maggiore Policlinico, Milan, Italy; and Dino Ferrari Center, University of Milan, Italy; Geriatric Unit, IRCCS Istituti Clinici Scientifici Maugeri, University of Milan, Italy; Department of Neurosciences and Mental Health, Fondazione IRCCS Cà Granda Ospedale Maggiore Policlinico, Milan, Italy; and Department of Pathophysiology and Transplantation, University of Milan, Italy; Neurodegenerative Diseases Unit, Fondazione IRCCS Ca' Granda Ospedale Maggiore Policlinico, Milan, Italy

**Keywords:** Diogenes syndrome, frontotemporal dementia, hoarding, collecting

## Abstract

**Background:**

Diogenes syndrome is a neurobehavioural syndrome characterised by domestic squalor, hoarding and lack of insight. It is an uncommon but high-mortality condition, often associated with dementia.

**Aims:**

To describe the clinical features and treatment of Diogenes syndrome secondary to behavioural variant frontotemporal dementia (bvFTD).

**Method:**

We describe a case of bvFTD in a 77-year-old man presenting with Diogenes syndrome.

**Results:**

The patient's medical and psychiatric histories were unremarkable, but in recent years he had begun packing his flat with ‘art pieces’. Mental state examination revealed confabulation and more structured delusions. Neuropsychological evaluation outlined an impairment in selective attention and letter verbal fluency, but no semantic impairment, in the context of an overall preserved mental functioning. Brain magnetic resonance imaging and positron emission tomography (PET) with fluorodeoxyglucose showed mild bilateral temporo-insular atrophy and hypometabolism in the left-superior temporal gyrus respectively. An amyloid PET scan and genetic analysis covering the dementia spectrum were normal. A diagnosis of bvFTD was made.

**Conclusions:**

The clinical framing of behavioural symptoms of dementia such as hoarding poses a diagnostic challenge. This case illustrates the importance of a deeper understanding of Diogenes syndrome, leading to timelier diagnosis and effective therapeutic strategies.

Diogenes syndrome – named after the Greek philosopher and cynic – is a neurobehavioural syndrome characterised by severe domestic squalor, pathological hoarding and lack of insight into the condition,^1^ the latter preventing the majority of patients from seeking medical help. First cases of elderly patients with self-neglect and extreme lack of hygiene were published by Dupré in 1925^2^ and Stevens in 1963^3^ and then more precisely described as a syndrome in 1966 by Macmillan & Shaw,^4^ who called this condition ‘senile breakdown’. Halliday et al in 2000 proposed domestic squalor, evidence of self-neglect, living alone, tendency to hoard and lack of concern for surroundings as the five defining features of the syndrome and they also developed the Environmental Cleanliness and Clutter Scale to assess squalor and hoarding.^5^ However, even in their study only 22% of individuals met all items on the scale and diagnostic criteria still lack consensus.^5^

The majority of cases occur in older adults (average age of 79 years) who live alone, although rare cases have been described in siblings and married couples.^6^ The current incidence of the full syndrome is not well-known: a retrospective observational French study found 1.6 cases per 10 000 inhabitants; 25% of patients had the complete syndrome and 75% had the partial syndrome.^7^ Cipriani et al estimate an approximate annual incidence of 0.05% in people over the age of 60.^8^ In all probability, our society will be faced with more cases in the future owing to increased life expectancy and a consequent higher number of elderly individuals living alone.

Despite being an uncommon condition, diagnosis is paramount since Diogenes syndrome has been associated with increased morbidity and a 46% 5-year mortality rate,^9^ with death commonly due to physical illnesses subsequent to self-neglect. Moreover, Diogenes syndrome poses ethical questions and legal challenges, such as finding a balance between autonomy and beneficence.^10^

Although the syndrome may occur as a single entity – so-called primary Diogenes syndrome – it is usually secondary to an underlying neuropsychiatric condition. Overall, Diogenes syndrome tends to be associated with psychosis, alcohol-induced disorder, affective disorder or obsessive–compulsive disorder (OCD) in younger individuals, whereas it is frequently associated with dementia in the elderly: clinical hoarding behaviour, often associated with self-neglect, and Diogenes syndrome are described in 23%^11^ and 15%^12^ respectively of older individuals with dementia. The unique combination of behavioural symptoms of behavioural variant frontotemporal dementia (bvFTD) may predispose to a high likelihood of developing Diogenes syndrome, which in fact occurs in up to 36% of people with bvFTD.^13^ We describe here a case of bvFTD presenting with Diogenes syndrome and subsequent follow-up of the patient.

## Ethics statement

Informed written consent approved by the local Institutional Review Board was obtained from the patient, in accordance with specific national laws and the ethics standards laid down in the 1964 Declaration of Helsinki and its later amendments.

## Case report

**Fig. 1:**
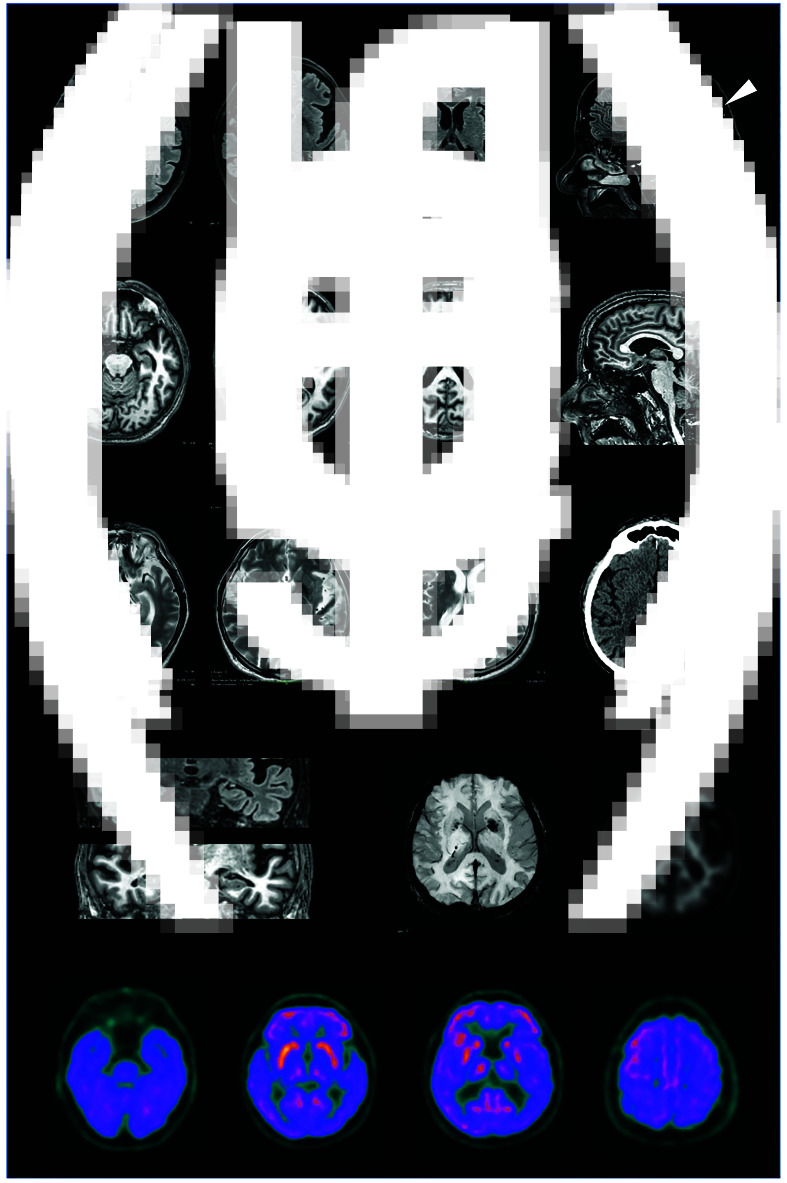
Brain scans showing mild temporo-insular atrophy on both sides, calcifications in the left lenticular nucleus and the location of the previously removed meningioma (arrowhead). (a) Fluid attenuated inversion recovery (FLAIR) and (b) T_1_-weighted axial and sagittal (rightmost images) scans. (c) T_2_-weighted axial scans. (d) Computed tomography axial scan. (e) Coronal FLAIR section of temporal lobes (above) and coronal T_1_ scans of temporal lobes (below). (f) Susceptibility weighted imaging (SWI) scan. (g) Amyloid positron emission tomography (PET) scan, negative for amyloid deposition. (h) fluorodeoxyglucose PET scans showing left superior temporal hypometabolism.

A 77-year-old dextral man was referred to the emergency department of our hospital by the police because he was found on the landing outside his flat, unkempt and dishevelled in personal appearance, after reportedly having lived there for 10 days. When questioned about this behaviour, he claimed that he was waiting for his next-door neighbour, who held a spare set of his flat keys, since he had accidentally locked his set in his garage. However, while he believed her to be on holiday, she had actually been living in a retirement home for 2 years. His medical history was significant only for a craniotomy, performed 16 years before, for the excision of a left parietal meningioma, with no residual neurological deficits, and hypertension, for which he did not take any drugs. He admittedly consumed more than a litre of wine per day but denied smoking. His psychiatric history was unremarkable, but in recent years he had begun packing his flat with ‘art pieces’. An electrocardiogram performed in the emergency department revealed the presence of atrial fibrillation, and a chest radiography showed a right lower lobe consolidation, which were treated with oral medication because he refused any infusion therapy. He was initially admitted to the geriatric department and then moved to the psychiatric unit because of repeated instances of wandering and a quarrel with another homeless in-patient. When contacted, the patient's building manager reported behavioural changes over the past few years, and a recent history of pathological hoarding was confirmed by his general practitioner. A mental state examination revealed confabulation, along with a more structured delusion about being married to a counterterrorism spy, daughter of a 19th-century marshal, with whom he would communicate only non-verbally in order for her not to be discovered. Neuropsychological evaluation outlined an impairment in selective attention and letter verbal fluency retrieval, in the context of an overall preserved mental functioning, with a score of 26/30 on the Mini-Mental State Examination (MMSE); a subsequent more thorough testing for language excluded semantic impairment (Table 1). Brain magnetic resonance imaging (MRI) showed a mild temporo-insular atrophy on both sides and calcifications in the left lenticular nucleus; fluorodeoxyglucose positron emission tomography (FDG-PET) revealed mild hypometabolism in the left superior temporal gyrus; amyloid PET did not reveal areas of increased radiotracer binding, ruling out an underlying amyloid brain deposition (Fig. 1). The search for expansion in the Chromosome 9 open reading frame 72 (*C9orf72*) gene gave negative results, and no mutations were detected through next generation sequencing (NGS) analysis.^14^ According to Rascovsky criteria,^15^ a diagnosis of bvFTD was made. In view of agitation and delusions, the patient was initially prescribed clotiapine, then shifted to zuclopenthixol and eventually to haloperidol, with only partial response. After the hospital admission, the patient was admitted to a care home.

**Table 1:** Neuropsychological evaluation scores

Cognitive function and test	Reference value	Raw score	Corrected score	Interpretation
Global cognitive functioning				
Mini-Mental State Examination (MMSE)	>23.8	26/30	28.03/30	Normal
Attention				
Attentional Matrices	>29.9	31/60	34.25/60	Inferior limit
Trail Making Test (part A)	<93	50	19	Normal
Working memory				
Letter verbal fluency	>17.35	4	13.4	Impaired
Language				
Sartori Naming Test	>50.36	62/64	37	Normal
Semantic fluency	>25	27		Normal
Semantic association (intracategorical)	>17.92	18/20		Inferior limit
Anterograde episodic memory				
Short Story Task	>4.75	10/16	10.75/16	Normal
Visuospatial function				
Clock Drawing Test	>6.55	13/13	13/13	Normal
Constructive apraxia				
Rey Figure Copy	>28.8	30/36	32.75/36	Normal

## Discussion

The exact pathogenesis and neuroanatomical basis of the full Diogenes syndrome are only hypothetical. In fact, no definite triggers to the condition have been identified and it is not clear whether solitude is a risk factor or a consequence of decision-making. Also, while hoarding is a major feature of Diogenes syndrome, there is no agreement on how to consider it in respect to other manifestations of the full syndrome (namely, squalor), to possibly related obsessive–compulsive behaviours and to other nosological entities such as OCD. The DSM-5 considers hoarding disorder and OCD to be distinct clinically, neuroanatomically and therapeutically. Collecting in OCD is associated with anxiety and fear and often driven by obsessions or preoccupations, whereas hoarding disorder is characterised by distress on discarding possessions, leading to clutter. Results from voxel-based morphometry studies have detected significant structural differences both in people with OCD compared with controls^16,17^ and in people with hoarding disorder relative to those with OCD and to controls.^18^ Also, Hough et al found significantly greater dorsolateral prefrontal cortex activation during executive tasks in individuals with hoarding disorder compared with those with OCD.^19^

In contrast to hoarding disorder, *acquired* hoarding has been described both as a consequence of vascular lesions in the medial prefrontal or bilateral orbitofrontal cortex^20^ and in the context of dementia.^21^ Despite not being specific to any dementia subtype, hoarding appears to be much more common in bvFTD and semantic variant primary progressive aphasia (svPPA) – subtypes of FTD – occurring both in isolation and in the presence of additional obsessive–compulsive behaviours.^22^

Various explanations have been offered to account for the new onset of compulsive collecting in individuals with dementia.

Frontal lobe deficits are common in all the FTD subtypes and could clearly contribute to increased clutter due to a combination of disturbances. Finney et al reviewed five cases of Diogenes syndrome in individuals with bvFTD who exhibited hoarding accompanied by decline in self-care, other obsessive–compulsive behaviours and prominent executive dysfunctions.^23^ All these patients had bilateral or right predominant frontotemporal atrophy and hypometabolism, suggesting that compulsive collecting in these patients may be part of the environmental dependency syndrome in frontal disease, with specific involvement of a right fronto-limbic-striatal system.

On the other hand, results from a quantitative MRI volumetric analysis of 47 individuals with FTD showed hoarding and obsessive–compulsive behaviours to be partially dissociable clinically and neuroanatomically, though highlighting an important role of the temporal lobes, particularly the left, in both disturbances.^22^ The study found that new or increased hoarding behaviour in people with FTD were more frequently associated with svPPA and predicted by left temporal atrophy. Left lateral temporal lobe grey matter loss also correlated with severity of obsessive–compulsive behaviours in a study conducted by Perry et al on 11 people with bvFTD.^21^ A plausible explanation for this finding is that deterioration of semantic organisation and storage secondary to degeneration of left temporal lobe structures, particularly in svPPA, may impair categorisation of objects, causing each to be considered unique and therefore non-discardable. Interestingly, in line with these newer data, our patient showed mild left superior temporal gyrus hypometabolism on the FDG-PET scan, much more limited in respect to the extensive frontotemporal hypometabolism in Finney et al's series^23^ and usually observed in bvFTD.^24^ However, to the best of our knowledge no previous studies specifically aimed at comparing patterns of cerebral glucose metabolism in individuals with FTD with and without hoarding are available to support our finding.

In conclusion, even if the development of the full Diogenes syndrome may require a more extensive and predominantly right-sided impairment of frontotemporal lobe connectivity and function, as seen in bvFTD, the occurrence of hoarding as an isolated finding in people with dementia, particularly in svPPA, along with newer literature suggests a pivotal role of left temporal lobe pathology in compulsive collecting, which our case seems to confirm.

Therapeutic management of Diogenes syndrome includes non-pharmacological and pharmacological approaches. Overall, both have proven difficult, since most patients deny their condition, avoid medical attention and exhibit poor therapeutic adherence.

Behavioural therapy-based skills seem beneficial.^25^ However, owing to the absence of randomised controlled trials, there are no guidelines on pharmacological interventions and no approved drugs, so that pharmacological interventions frequently depend on associated symptoms when present.

Many drugs have been tried in different studies, including selective serotonin reuptake inhibitors (SSRIs), first- and second-generation antipsychotics and anti-epileptics, with mixed results. Given the well acknowledged role of serotonin and dopamine in impulse control disorders and the approved use of SSRIs in the treatment of OCD, a therapeutic trial with molecules from one of these classes may be justified.

In fact, Finney et al reported some effect of high-dose sertraline in reducing collecting behaviours in two of their patients.^23^ Other authors found benefit on hoarding from other SSRIs^26^ and a combination of valproic acid and quetiapine in treating patients with bvFTD and secondary Diogenes syndrome.^27^ Lithium significantly improved one patient with long-standing Diogenes syndrome, although probably secondary to bipolar disorder,^28^ and risperidone reduced hoarding in an individual with Diogenes syndrome, but caused significant motor side-effects.^29^ Although not specifically tried for hoarding, trazodone has proven beneficial for behavioural symptoms in FTD.^30^

Our patient needed typical antipsychotics to manage agitation but quitting hoarding was mainly due to hospital admission and no specific treatment was tried. Admitting patients to hospital or moving them to another location is sometimes mandatory, but out-patient treatment through community care should be privileged if there is little risk to the patient or neighbours. Management should be conducted sensitively lest patients return to previous living conditions even more reluctant to receive medical aid.

### Implications

This case, while illustrating the importance of suspecting Diogenes syndrome in elderly patients presenting with squalor and hoarding, especially in the context of dementia, supports recent evidence about the importance of the left temporal lobe in compulsive collecting pathogenesis. A deeper understanding of this condition, facilitating a timelier diagnosis, may lead to more effective pharmacological and non-pharmacological interventions, which in turn may reduce acute and chronic physical illness and improve social health outcomes.

## Data Availability

The study data are available on reasonable request.

## References

[1] Assal F. Diogenes syndrome. Front Neurol Neurosci 2018; 41, 90–7. 29145187 10.1159/000475688

[2] Dupré E. Les mendiants thésauriseurs. [The beggars hoarders.] In Pathologie de *L'imagination L’émotivité*. Payot, 1925: 429–44.

[3] Stevens RS. Self-neglect in the elderly. Br J Geriatr Pract 1963; 2: 88–91.

[4] Macmillan D, Shaw P. Senile breakdown in standards of personal and environmental cleanliness. BMJ 1966; 2: 1032–7. 5919035 10.1136/bmj.2.5521.1032 PMC1944569

[5] Halliday G, Banerjee S, Philpot M, Macdonald A. Community study of people who live in squalor. Lancet 2000; 355: 882–6. 10752704 10.1016/S0140-6736(99)06250-9

[6] Halliday G, Snowdon J, Simpson B. Re: Diogenes syndrome in a pair of siblings. Can J Psychiatry 2005; 50: 567. 10.1177/070674370505000914 16262113

[7] Monfort J-C, Hugonot-Diener L, Devouche E, Wong C, Péan I. Le syndrome de Diogène et les situations apparentées d'auto-exclusion sociale: enquête descriptive. [Diogenes syndrome and related situations of social self-exclusion: descriptive survey.] Psychol Neuropsychiatr Vieil 2010; 8: 141–53. 20525545 10.1684/pnv.2010.0215

[8] Cipriani G, Lucetti C, Vedovello M, Nuti A. Diogenes syndrome in patients suffering from dementia. Dialogues Clin Neurosci 2012; 14: 455–60. 23393422 10.31887/DCNS.2012.14.4/gcipriani PMC3553571

[9] Clark ANG, Mankikar GD, Gray I. Diogenes syndrome. Lancet 1975; 305: 366–8. 10.1016/s0140-6736(75)91280-5 46514

[10] Freckelton I. Hoarding disorder and the law. J Law Med 2012; 20: 225–49. 23431842

[11] Hwang JP, Tsai SJ, Yang CH, Liu KM, Lirng JF. Hoarding behavior in dementia: a preliminary report. Am J Geriatr Psychiatry 1998; 6: 285–9. 9793576

[12] Radebaugh TS, Hooper FJ, Gruenberg EM. The social breakdown syndrome in the elderly population living in the community: the Helping Study. Br J Psychiatry 1987; 151: 341–6. 3501323 10.1192/bjp.151.3.341

[13] Lebert F. Diogene syndrome, a clinical presentation of fronto-temporal dementia or not? Int J Geriatr Psychiatry 2005; 20: 1203–4. 16315145 10.1002/gps.1430

[14] Ghezzi L, Carandini T, Arighi A, Fenoglio C, Arcaro M, De Riz M, Evidence of CNS β-amyloid deposition in Nasu-Hakola disease due to the *TREM2* Q33X mutation. Neurology 2017; 89: 2503–5. 29142083 10.1212/WNL.0000000000004747

[15] Rascovsky K, Hodges JR, Knopman D, Mendez MF, Kramer JH, Neuhaus J, Sensitivity of revised diagnostic criteria for the behavioural variant of frontotemporal dementia. Brain 2011; 134: 2456–77. 21810890 10.1093/brain/awr179 PMC3170532

[16] Piras F, Piras F, Chiapponi C, Girardi P, Caltagirone C, Spalletta G. Widespread structural brain changes in OCD: a systematic review of voxel-based morphometry studies. Cortex 2015; 62: 89–108. 23582297 10.1016/j.cortex.2013.01.016

[17] Subirà M, Cano M, de Wit SJ, Alonso P, Cardoner N, Hoexter MQ, Structural covariance of neostriatal and limbic regions in patients with obsessive–compulsive disorder. J Psychiatry Neurosci 2016; 41: 115–23. 26505142 10.1503/jpn.150012 PMC4764480

[18] Yamada S, Nakao T, Ikari K, Kuwano M, Murayama K, Tomiyama H, A unique increase in prefrontal gray matter volume in hoarding disorder compared to obsessive-compulsive disorder. PloS One 2018; 13: e0200814. 30011337 10.1371/journal.pone.0200814 PMC6047811

[19] Hough CM, Luks TL, Lai K, Vigil O, Guillory S, Nongpiur A, Comparison of brain activation patterns during executive function tasks in hoarding disorder and non-hoarding OCD. Psychiatry Res Neuroimaging 2016; 255: 50–9. 27522332 10.1016/j.pscychresns.2016.07.007 PMC5014569

[20] Figee M, Wielaard I, Mazaheri A, Denys D. Neurosurgical targets for compulsivity: what can we learn from acquired brain lesions? Neurosci Biobehav Rev 2013; 37: 328–39. 23313647 10.1016/j.neubiorev.2013.01.005

[21] Perry DC, Whitwell JL, Boeve BF, Pankratz VS, Knopman DS, Petersen RC, Voxel-based morphometry in patients with obsessive-compulsive behaviors in behavioral variant frontotemporal dementia: compulsions in FTD. Eur J Neurol 2012; 19: 911–7. 22284815 10.1111/j.1468-1331.2011.03656.x PMC3351534

[22] Mitchell E, Tavares TP, Palaniyappan L, Finger EC. Hoarding and obsessive–compulsive behaviours in frontotemporal dementia: clinical and neuroanatomic associations. Cortex 2019; 121: 443–53. 31715541 10.1016/j.cortex.2019.09.012

[23] Finney CM, Mendez MF. Diogenes syndrome in frontotemporal dementia. Am J Alzheimers Dis Other Demen 2017; 32: 438–43. 28660777 10.1177/1533317517717012 PMC10852732

[24] Cerami C, Dodich A, Lettieri G, Iannaccone S, Magnani G, Marcone A, Different FDG-PET metabolic patterns at single-subject level in the behavioral variant of fronto-temporal dementia. Cortex 2016; 83: 101–12. 27498041 10.1016/j.cortex.2016.07.008

[25] Waserman JE, Hategan A, Bourgeois JA. Harnessing neuroplasticity in Diogenes syndrome: a proposed mechanism to explain clinical improvement. Gen Hosp Psychiatry 2014; 36: 761.e3–e5. 10.1016/j.genhosppsych.2014.06.013 25091139

[26] Saxena S, Brody AL, Maidment KM, Baxter LR. Paroxetine treatment of compulsive hoarding. J Psychiatr Res 2007; 41: 481–7. 16790250 10.1016/j.jpsychires.2006.05.001 PMC2876089

[27] Gálvez-Andres A, Blasco-Fontecilla H, González-Parra S, Molina JD, Padín JM, Rodriguez RH. Secondary bipolar disorder and Diogenes syndrome in frontotemporal dementia: behavioral improvement with quetiapine and sodium valproate. J Clin Psychopharmacol 2007; 27: 722–3. 18004150 10.1097/JCP.0b013e31815a57c1

[28] Fond G, Jollant F, Abbar M. The need to consider mood disorders, and especially chronic mania, in cases of Diogenes syndrome (squalor syndrome). Int Psychogeriatr 2011; 23: 505–7. 20836916 10.1017/S1041610210001663

[29] Herrán A, Vázquez-Barquero JL. Treatment of Diogenes syndrome with risperidone. Aging Neuropsychol Cogn 1999; 6: 96–8.

[30] Lebert F, Stekke W, Hasenbroekx C, Pasquier F. Frontotemporal dementia: a randomised, controlled trial with trazodone. Dement Geriatr Cogn Disord 2004; 17: 355–9. 15178953 10.1159/000077171

